# Endovascular Treatment of Stroke and Anesthesia Technique: What Is the Best Approach, According to the Literature?

**DOI:** 10.3390/neurolint17080115

**Published:** 2025-07-25

**Authors:** Federica Arturi, Gabriele Melegari, Fabio Gazzotti, Elisabetta Bertellini, Alberto Barbieri

**Affiliations:** 1 School of Anaesthesia and Intensive Care, University of Modena and Reggio Emilia, Via del Pozzo 71, 4112 Modena, Italy; federica.arturi.94@gmail.com (F.A.); gazzottifabio@gmail.com (F.G.); 2 Department of Anaesthesia and Intensive Care, Azienda Ospedaliera Universitaria di Modena, Via del Pozzo 71, 4112 Modena, Italy; 3 School of Medicine, University of Modena and Reggio Emilia, Via del Pozzo 71, 4112 Modena, Italy; elisabettab53@gmail.com

**Keywords:** stroke, endovascular thrombectomy, general anesthesia, conscious sedation, local anesthesia, neurological outcome, blood pressure management, infection risk, ischemic stroke, perioperative care

## Abstract

Background/Objectives: Endovascular thrombectomy has become a mainstay in the treatment of acute ischemic stroke caused by large vessel occlusion. Among the multiple factors that influence outcomes, the choice of anesthetic technique—general anesthesia (GA), conscious sedation (CS), or local anesthesia (LA)—remains controversial. This narrative review aims to critically examine and synthesize current evidence comparing the efficacy and safety of different anesthetic strategies in endovascular stroke treatment. Methods: A structured search of the PubMed^®^ database was conducted using the terms “stroke treatment”, “endovascular stroke treatment”, “anesthesia”, “general anesthesia”, “conscious sedation”, and “local anesthesia”. The search focused on clinical trials involving human subjects published in English. Studies were included if they compared at least two anesthetic techniques during thrombectomy and reported outcomes such as neurological recovery, mortality, or complication rates. Reviews, case reports, and animal studies were excluded. Results: Several randomized controlled trials and observational studies show comparable functional outcomes between GA and CS, though CS may confer advantages in early neurological recovery and reduced complications. Local anesthesia, though less studied, may offer favorable outcomes in selected patients. General anesthesia appears to be associated with greater hemodynamic variability and a higher risk of post-procedural infections, particularly in unsuccessful interventions. Maintaining stable blood pressure and minimizing ventilation duration are crucial to improving patient prognosis. Conclusions: While both GA and CS are viable options during thrombectomy, CS and LA may provide a safer profile in selected patients by preserving hemodynamic stability and reducing infectious risk. Personalized anesthetic strategies and further high-quality trials are warranted.

## 1. Introduction

Endovascular thrombectomy has emerged as a standard of care for patients with large vessel occlusion, ideally performed within 6 h—and up to 24 h in selected cases—following advanced imaging assessment [1,2,3]. Given the time-sensitive nature of these interventions, streamlining procedural logistics and peri-procedural care is critical to optimizing clinical outcomes. Among the variables influencing procedural success and patient safety, the choice of anesthetic technique during endovascular thrombectomy plays a pivotal role. The anesthetic approach must ensure absolute patient immobility to facilitate the procedure but also preserve hemodynamic stability and allow, when appropriate, continuous neurological monitoring. Available options include general anesthesia (GA), conscious sedation (CS), or, in limited cases, local anesthesia at the vascular access site, with or without adjunctive sedation. Each modality carries its own set of advantages and limitations. General anesthesia provides a controlled airway, optimal patient immobility, and complete pain relief, which can facilitate procedural performance. However, it may be associated with delayed initiation of thrombectomy, potential hypotension, and suppression of neurological assessment during the procedure. Conversely, conscious sedation enables faster workflow, allows real-time neurological evaluation, and is generally associated with greater hemodynamic stability. Nevertheless, inadequate sedation or patient movement can compromise procedural safety and success [4,5,6]. In light of these factors, numerous studies and randomized trials have been conducted to compare the clinical outcomes associated with general anesthesia versus conscious sedation in the setting of acute ischemic stroke. However, the literature has yielded heterogeneous and, at times, conflicting results, with some studies favoring conscious sedation due to better functional outcomes and fewer complications, while others suggest no significant difference or even improved outcomes with general anesthesia.

The objective of this review is to provide a comprehensive and critical synthesis of the available evidence comparing anesthetic techniques during endovascular treatment of ischemic stroke. Our aim is to offer healthcare professionals an accessible and practical summary of the current data to support informed decision-making in the acute care of these complex patients.

## 2. Materials and Methods

This narrative review was conducted in accordance with the most recent version (2019) of the SANRA (Scale for the Assessment of Narrative Review Articles) guidelines, which provide a standardized framework for assessing the methodological quality of non-systematic reviews in the biomedical literature [7]. The aim was to ensure clarity, methodological rigor, and relevance in the selection and synthesis of the scientific evidence included in the review.

The literature search was carried out using the PubMed^®^ database, chosen for its comprehensive coverage of peer-reviewed biomedical research. To maximize the sensitivity and specificity of the search, a combination of relevant Medical Subject Headings (MeSH) terms and free-text keywords was used. Boolean operators AND, OR, and NOT were applied to build a structured search strategy. The search terms included: “stroke treatment”, “endovascular stroke treatment”, “anesthesia”, “general anesthesia”, “conscious sedation”, and “local anesthesia”. Searches were limited to studies published in English and focused specifically on clinical research involving human subjects. Inclusion criteria were as follows: clinical trials (both randomized and observational) evaluating the impact of anesthetic technique on outcomes in patients undergoing endovascular treatment for acute ischemic stroke. Studies comparing general anesthesia and conscious sedation, or including data on local anesthesia during endovascular procedures. Studies with clearly defined outcomes such as recanalization rate, functional neurological outcome, mortality, or procedural complications. Review articles, case reports, editorials, animal studies, and studies not directly relevant to the core objective of this review were excluded. In addition, systematic reviews and meta-analyses already indexed in PubMed were not included as primary sources, although their reference lists were examined for additional eligible studies not captured in the initial search. The initial screening was performed by two independent reviewers who assessed titles and abstracts for relevance. Full-text screening was then conducted for all selected articles. Discrepancies were resolved by discussion among the authors. The final dataset of articles was analyzed qualitatively, focusing on study design, patient population, anesthetic approach, and reported outcomes. The goal was to provide an up-to-date and balanced synthesis of the most relevant clinical evidence to inform anesthetic management decisions during endovascular therapy for acute ischemic stroke.

## 3. Comparison Between General Anesthesia and Conscious Sedation

The choice between general anesthesia (GA) and conscious sedation (CS) in patients undergoing endovascular treatment for acute ischemic stroke has been the subject of growing interest and research in recent years. This debate is clinically relevant because anesthesia technique may impact time to reperfusion, hemodynamic stability, neurological assessment during the procedure, and ultimately functional outcome and mortality. Several clinical trials have specifically addressed this comparison, yielding at times divergent or inconclusive findings. Table 1 summarizes the main characteristics and outcomes of the most representative studies available to date.

One of the earliest randomized controlled trials in this field is the SIESTA trial by Schönenberger et al., which prospectively compared GA and CS in patients undergoing mechanical thrombectomy. Patients in the GA group underwent orotracheal intubation, whereas those in the CS group maintained spontaneous breathing. Neurological outcomes were assessed at 24 h and 90 days using standardized tools such as the NIH Stroke Scale (NIHSS) and the modified Rankin Scale (mRS). The trial demonstrated a favorable trend toward better safety profiles and neurological outcomes in the CS group, particularly regarding early post-procedural assessment [4].

In contrast, the AMETIS trial (Chabanne et al.) examined differences in functional outcome and periprocedural complications in patients with anterior circulation stroke treated with either GA or CS. Their results, however, showed no statistically significant differences between the two groups in terms of 90-day mRS or complication rates, suggesting comparable safety and efficacy profiles for both anesthetic strategies in this context [8].

A similar conclusion was reached by Just et al., who conducted a large-scale multicenter study in France comparing mortality and morbidity (assessed by mRS at 3 and 6 months) between the two anesthetic techniques. Their results supported the SIESTA findings, indicating that patients managed with GA experienced higher mortality and worse long-term neurological outcomes compared to those treated with CS [9].

On the other hand, Liang et al. specifically addressed the less commonly studied subset of patients with posterior circulation strokes. Their findings showed no significant differences in neurological outcomes, procedural time, or hospital stay between the two techniques. Interestingly, they noted a high rate of conversion from CS to GA, supporting GA as the potentially safer initial choice in these anatomically and clinically complex cases [10]. Another study by Simonsen et al. focused on radiological endpoints, particularly infarct volume growth on MRI. Their analysis found no significant difference in infarct progression between the GA and CS groups, indicating that anesthetic technique did not influence the extent of ischemic damage in the early post-procedural phase [11]. The GASS Randomized Trial, conducted by Maurice et al., further confirmed the non-inferiority of CS versus GA. This study demonstrated similar rates of neurological recovery and functional independence at 3 months, reinforcing the feasibility of using either technique based on institutional experience and patient condition [12]. Comparable conclusions were drawn in the CANVAS trial by Sun et al., which reported similar outcomes regarding revascularization success and neurological function recovery at 90 days. However, this study, like Liang’s, highlighted the risk of emergency conversion from CS to GA, particularly in patients who exhibited insufficient immobility or respiratory compromise under sedation. This remains a critical safety consideration for procedural planning [13].

A number of other trials and meta-analyses have corroborated these findings, suggesting that the overall impact of anesthetic technique on mortality and long-term neurological outcomes may be limited, and that differences are often attributable to patient selection, timing, and operator preference rather than the technique per se [14,15,16].

Of note, Gaspari et al. proposed a structured internal protocol for the standardized use of conscious sedation, aimed at reducing variability and improving patient safety. Their protocol is based on continuous infusion of propofol, titrated to achieve adequate sedation while preserving spontaneous ventilation. In cases where sedation alone is insufficient, remifentanil can be added as a secondary agent, or additional propofol boluses can be administered. This structured approach may help mitigate the risk of conversion to GA and improve the consistency of outcomes with CS [17]. A recent retrospective multicenter matched analysis by Valente et al. provided valuable insight into anesthetic management for patients with minor stroke and isolated M2 occlusion undergoing immediate thrombectomy. This specific patient population is frequently underrepresented in randomized controlled trials. The study compared outcomes among patients treated under general anesthesia, conscious sedation, or local anesthesia. Findings indicated that both conscious sedation and local anesthesia were associated with better 90-day functional outcomes compared to general anesthesia, without a significant increase in procedural complications. These results support the notion that less invasive anesthetic strategies may offer clinical advantages in selected patients with mild neurological deficits and straightforward vascular access, by preserving physiological stability and allowing timely neurological assessment [18].

In conclusion, while early studies seemed to favor conscious sedation for its safety and neurological advantages, more recent randomized trials and meta-analyses suggest that both GA and CS can be used effectively during endovascular thrombectomy, provided they are applied within standardized protocols and tailored to patient-specific clinical features. The decision should, therefore, be based on institutional expertise, anesthesiologist availability, patient cooperation, airway risk, and procedural complexity.

**Table 1:** Summary of clinical trials comparing GA and CS.

Title	Authors	Year of Publication	Superiority of GA or CS	Evaluated Outcomes
SIESTA randomized monocentric trial	Schönenberger et al. [4]	2015	CS > GA	NIHSS after 24 h, mRS after 3 months, procedural complications, mortality
AMETIS Randomized Clinical Trial	Chabanne et al. [8]	2023	CS = GA	Procedural complications, functional outcome
Outcomes of General Anesthesia and Conscious Sedation in Endovascular Treatment for Stroke	Just et al. [9]	2016	CS > GA	mRS scale after 3 and 6 months, mortality
General Anesthesia vs. Conscious Sedation for Endovascular Treatment in Patients with Posterior Circulation Acute Ischemic Stroke: An Exploratory Randomized Clinical Trial	Liang et al. [10]	2022	CS = GA	(mRS at 3 months), mortality, procedure duration, and hospital length of stay
Effect of General Anesthesia and Conscious Sedation During Endovascular Therapy on Infarct Growth and Clinical Outcomes in Acute Ischemic Stroke: A Randomized Clinical Trial	Simonsen et al. [11]	2018	CS = GA	infarct growth volume
okGASS Randomized Trial	Maurice et al. [12]	2022	CS = GA	mRS at 3 months
CANVAS trial	Sun et al. [13]	2020	CS = GA	mRS at 3 months, success rate of the revascularization procedure
Anesthesia for Endovascular Therapy for Stroke	Gaspari et al. [17]	2024	CS = GA	NIHSS score at 7 days, mRS at 3 months

## 4. Comparison with Local Anesthesia

While most of the literature comparing anesthetic techniques in endovascular stroke treatment has focused on general anesthesia (GA) and conscious sedation (CS), a smaller but noteworthy body of evidence has also explored the role of local anesthesia (LA)—with or without minimal sedation—as a viable alternative. This approach, although less commonly adopted in routine clinical practice, deserves attention, particularly in light of efforts to optimize procedural efficiency, reduce risks associated with sedation or airway manipulation, and preserve neurological assessment. Van den Graaf et al. investigated this aspect by comparing outcomes in patients with acute ischemic stroke who were managed either with local anesthesia alone or with conscious sedation during endovascular thrombectomy [19]. Their findings suggest that local anesthesia may offer advantages in terms of safety and functional outcomes. Specifically, patients treated with LA demonstrated better neurological recovery and lower mortality rates, without an increase in periprocedural complications. The authors also noted that the use of conscious sedation did not confer additional benefits in terms of reducing procedure duration or complication rates. These results challenge the assumption that sedation is always necessary and suggest that, in selected patients, LA could be both safe and effective [15]. Further evidence supporting the safety profile of local anesthesia comes from the study by Wu et al., which focused on patients with posterior circulation stroke—a subgroup known for its high clinical severity and frequent need for rapid intervention. In this study, neurological outcomes and mortality rates were compared between patients undergoing revascularization under GA and those treated with LA. The results revealed no significant difference between the two groups, suggesting that the choice of anesthetic technique may not substantially impact revascularization success or overall prognosis in this specific clinical context [16]. These findings are particularly relevant when considering certain logistical or patient-specific factors. For instance, local anesthesia avoids the risks associated with airway instrumentation, such as aspiration or hypoventilation, and may allow for faster procedural initiation in emergency settings. It also maintains full patient cooperation, which can be valuable when neurological monitoring during the intervention is desired. Nevertheless, the use of local anesthesia requires strict patient selection, given the potential for anxiety, pain, or movement during the procedure, which could compromise both technical success and patient safety. Moreover, the need for conversion to GA must always be anticipated and prepared for, especially in patients with fluctuating consciousness, agitation, or respiratory compromise. In summary, although underrepresented in major trials, local anesthesia represents a promising and potentially underutilized strategy in selected cases of endovascular treatment for stroke. It offers favorable safety and outcome profiles and may be considered as a valid alternative, especially when rapid access, minimal physiological disruption, and preserved neurological monitoring are desired.

## 5. Hemodynamic Management and Infection Risk Across Techniques

Effective blood pressure (BP) management during endovascular revascularization is of critical importance for preserving the viability of the ischemic penumbra and preventing reperfusion injury. The ischemic penumbra represents a metabolically impaired but still salvageable region of brain tissue surrounding the infarct core. A key threat to this region is hypotension during or after recanalization, which can precipitate the conversion of potentially viable tissue into irreversible infarction due to insufficient cerebral perfusion [20,21].

Equally important is the opposite risk—hypertensive episodes following reperfusion, which may overwhelm the already compromised autoregulatory capacity of the cerebral vasculature. This breakdown in autoregulation can lead to cerebral hyperperfusion syndrome, a phenomenon associated with vasogenic edema, intracerebral hemorrhage, and secondary neuronal injury [22]. These hemodynamic extremes underscore the narrow therapeutic window required for optimal cerebral perfusion pressure during the intervention.

A pivotal analysis conducted by Leonardi-Bee et al., based on data from the International Stroke Trial, identified both hypotension and hypertension as independent predictors of poor functional outcome following ischemic stroke treatment. Elevated blood pressure during the procedure was associated with increased risk of cerebral edema and hemorrhagic transformation, whereas low BP carried a heightened risk of cardiovascular complications, including coronary ischemia and cardiac arrest [23,24].

Consistent with these findings, the MR CLEAN trial, conducted by Mulder et al., revealed that patients who maintained systolic blood pressure (SBP) around 120 mmHg during thrombectomy had significantly better functional outcomes. These results highlight the crucial need for tight hemodynamic control throughout the intervention to optimize both cerebral perfusion and systemic stability [25].

However, achieving such precise blood pressure targets is technically more challenging under general anesthesia, which is inherently associated with hemodynamic variability. During anesthetic induction, vasodilatory effects of agents such as propofol or volatile anesthetics may cause profound hypotension, while direct laryngoscopy and endotracheal intubation may trigger sympathetic stimulation and dangerous hypertensive surges. Conscious sedation and local anesthesia, by avoiding airway instrumentation and preserving spontaneous ventilation, are often associated with greater hemodynamic stability, offering a potential clinical advantage in this regard [26].

Beyond hemodynamics, another factor influencing patient prognosis is the risk of post-procedural infections, particularly in the critical care setting. While earlier studies suggested no clear correlation between anesthetic technique and infection risk [27,28], recent evidence calls this into question. A 2024 study by Jiang et al., a prospective clinical trial, has provided compelling data indicating that general anesthesia is independently associated with a higher incidence of infectious complications following endovascular stroke treatment, especially in cases where the procedure is unsuccessful [29]. According to the authors, several factors may contribute to this increased risk. These include: a higher likelihood of prolonged orotracheal intubation under GA, increasing the risk of ventilator-associated pneumonia, greater neurological impairment due to peri-procedural hypotension or delayed reperfusion, necessitating longer ICU stays, immunosuppressive effects related to deep sedation and mechanical ventilation.

The findings emphasize that infection prevention is not only a postoperative concern but also tightly linked to intraoperative anesthetic choices. Therefore, minimizing unnecessary exposure to mechanical ventilation and favoring techniques that preserve neurological function and shorten ICU length of stay—such as CS or LA—may have beneficial implications for infection control and overall outcomes. In conclusion, maintaining hemodynamic stability and minimizing infection risk are both critical goals in the anesthetic management of stroke patients undergoing thrombectomy. The current evidence tends to support conscious sedation or local anesthesia as more physiologically conservative and potentially safer strategies in many clinical scenarios. However, the final choice must be individualized based on patient characteristics, institutional protocols, and the availability of experienced personnel.

## 6. Conclusions

His review aimed to provide clinicians with a practical and evidence-based reference to support anesthetic decision-making in patients undergoing endovascular treatment for acute ischemic stroke. Although the literature presents heterogeneous and sometimes conflicting results, a growing body of evidence supports the preferential use of conscious sedation (CS) or local anesthesia (LA) over general anesthesia (GA) in appropriately selected patients. General anesthesia, while providing optimal immobility and airway control, has been consistently associated with higher rates of intraoperative hemodynamic instability—particularly hypotension during induction and hypertensive responses to airway manipulation—as well as an increased incidence of postoperative infections, especially in cases requiring prolonged mechanical ventilation or extended ICU stay. These factors may negatively impact cerebral perfusion, delay neurological recovery, and worsen overall prognosis. In contrast, CS and LA have demonstrated a more favorable safety profile in numerous studies, offering greater hemodynamic stability, fewer respiratory complications, and earlier neurological assessment. As such, the use of CS or LA should be more strongly recommended whenever feasible, especially in patients with minor strokes, stable airways, and cooperative behavior. Given the significant implications for patient safety and outcome, institutional protocols should prioritize less invasive anesthetic approaches, and future research should focus on refining patient selection criteria. High-quality multicenter randomized trials remain essential to define the optimal anesthetic strategy, but current evidence already provides a compelling rationale for limiting the routine use of GA in endovascular stroke therapy.

## Data Availability

Not applicable.
